# Além do *Pacing* Ventricular: A Cardiomiopatia Atrial como Determinante da Evolução Clínica após Implante de Marca-passo?

**DOI:** 10.36660/abc.20260425

**Published:** 2026-07-02

**Authors:** Bianca Lopes Cunha, Helena Nogueira Brasil, Patrícia de Araújo Matias, Eduardo Arrais Rocha

**Affiliations:** 1 Universidade Federal do Ceará Fortaleza CE Brasil Universidade Federal do Ceará, Fortaleza, CE – Brasil; 2 Hospital de Messejana Fortaleza CE Brasil Hospital de Messejana, Fortaleza, CE – Brasil; 3 Centro de Arritmia do Ceará Fortaleza CE Brasil Centro de Arritmia do Ceará (CACE), Fortaleza, CE – Brasil; 4 Universidade Federal do Ceará Hospital Universitário Walter Cantídio Fortaleza CE Brasil Universidade Federal do Ceará Hospital Universitário Walter Cantídio, Fortaleza, CE – Brasil; 5 Unichristus Fortaleza CE Brasil Unichristus, Fortaleza, CE – Brasil

**Keywords:** Marca-Passo Artificial, Cardiomiopatias, Remodelação Ventricular

O estudo "Resultados Clínicos após Implante de Marca-Passo: Um Estudo Comparativo de 3 Anos entre Síndrome do Nó Sinusal e Bloqueio Atrioventricular"^1^ traz uma análise comparativa relevante entre pacientes com a doença do nó sinusal (DNS) e o bloqueio atrioventricular (BAV), submetidos a implante de marca-passo (MP) permanente, acompanhados por três anos. O principal mérito do trabalho reside na avaliação clínica e do remodelamento cardíaco, nas taxas de fibrilação atrial (FA), reinternações e mortalidade comparativamente nos dois grupos.

Entre os pontos fortes do estudo destacam-se o seguimento relativamente prolongado, com avaliação ecocardiográfica seriada, mostrando diferenças importantes entre os padrões de remodelamento cardíaco. Pacientes com DNS apresentaram aumento progressivo do volume atrial esquerdo (VAE) e do diâmetro diastólico final do ventrículo esquerdo (DDVE), além de piora significativa da regurgitação mitral (RM). Em contrapartida, os pacientes com BAV, mesmo com carga de estimulação ventricular acima de 90%, apresentaram relativa estabilidade nas dimensões atriais e ventriculares. Ambos os grupos tiveram leves reduções na fração de ejeção (FE), altas taxas de FA, internações e mortalidade, sem diferenças significativas nos grupos. Esses achados desafiam parcialmente o conceito clássico de que a estimulação ventricular direita isoladamente seria a principal responsável pelo remodelamento cardíaco.

O remodelamento cardíaco corresponde a alterações moleculares, celulares e intersticiais que alteram o tamanho, a forma e a função cardíaca após lesão ou sobrecarga crônica.^2^ Clinicamente, manifesta-se por dilatação das câmaras e deterioração da função sistólica, constituindo importante substrato para insuficiência cardíaca (IC) e arritmias.

O trabalho merece reconhecimento também por discutir potenciais diferenças prognósticas entre DNS e BAV. Enquanto a RM foi associada à mortalidade na DNS, no BAV os preditores foram doença arterial coronariana, uso de betabloqueadores e aumento do volume atrial esquerdo (VAE). Esses achados sugerem mecanismos fisiopatológicos distintos e reforçam a necessidade de rigoroso seguimento desses pacientes.

A elevada incidência de FA em ambas as populações, superior a 35% em três anos reforça as observações previamente demonstradas em estudos como ASSERT^3^ e MOST,^4^ nos quais os pacientes portadores de dispositivos cardíacos apresentaram alta incidência de arritmias atriais subclínicas e aumento subsequente do risco tromboembólico.

Entretanto, algumas limitações metodológicas importantes embora citadas no estudo precisam ser consideradas. O estudo foi retrospectivo, unicêntrico e observacional, o que reduz sua capacidade de estabelecer relações causais robustas. O número de pacientes foi relativamente pequeno, especialmente no grupo DNS, limitando análises multivariadas mais consistentes. Além disso, não houve avaliação detalhada da posição do eletrodo ventricular, da ocorrência de dissincronia ventricular ou atrioventricular.

A ausência de dados mais completos sobre a IC e a miocardiopatia relacionada à estimulação limitam a análise. Estudos clássicos como DAVID,^5^ MOST^4^ e BLOCK-HF^6^ já demonstraram que elevadas cargas de estimulação ventricular direita podem promover disfunção ventricular e aumento de hospitalizações por IC. Curiosamente, neste estudo, os pacientes com BAV e carga de *pacing* acima de 90% não apresentaram diferenças na FE e paradoxalmente tiveram menores alterações no remodelamento cardíaco. Tal observação pode refletir diferenças basais de substrato atrial e miocárdico, mas também pode decorrer de limitações estatísticas e de análises ecocardiográficas, considerando diferentes analisadores e ausência de revalidação dos dados ecocardiográficos, ausência de análise de strain atrial e ventricular, além de dados sobre as posições dos eletrodos ventriculares e atriais.

Os autores sugerem que a própria DNS poderia representar um marcador independente de remodelamento. Essa hipótese é biologicamente plausível, especialmente considerando o crescente reconhecimento da cardiomiopatia atrial como entidade clínica. Estudos recentes^7,8^ demonstram que a fibrose atrial, inflamação, degeneração do sistema de condução e alterações estruturais do átrio precedem tanto a FA quanto as manifestações da DNS. Nesse contexto, a cardiomiopatia atrial pode ter sido um dos principais determinantes fisiopatológicos das diferenças observadas entre os grupos deste estudo. Esses achados desafiam diretamente a noção de que a estimulação ventricular isoladamente seja o principal determinante de remodelamento adverso.

Esses achados dialogam diretamente com conceitos mais modernos de cardiomiopatia atrial, nos quais o átrio esquerdo deixa de ser visto apenas como marcador indireto de doença cardiovascular e passa a ser compreendido como componente ativo do processo fisiopatológico cardiovascular. Tal interpretação fortalece ainda mais o racional para estratégias precoces de monitorização ecocardiográfica, vigilância de FA subclínica e eventual adoção de modalidades de estimulação fisiológica atrial em pacientes com DNS.

Por outro lado, a programação de intervalos AV excessivamente prolongados na DNS, com o objetivo de evitar a estimulação ventricular em pacientes com condução atrioventricular já comprometida, pode trazer prejuízos hemodinâmicos, como RM diastólica, levando ao aparecimento de sintomas e/ou FA.^9^

Do ponto de vista clínico, o estudo reforça a necessidade de seguimento ecocardiográfico periódico dos pacientes com MP, particularmente daqueles com DNS. Assim, a porcentagem de *pacing* deve ser interpretada não apenas como marcador de dependência do dispositivo, mas como expressão da fisiopatologia subjacente. Enquanto no BAV, a preocupação tradicional permanece centrada na dissincronia ventricular induzida pelo *pacing,* na DNS o maior desafio parece ser a progressão silenciosa da doença atrial e ventricular estrutural.

Dessa forma, os achados do estudo e as diretrizes contemporâneas^9,10^ sugerem que o seguimento pós-implante deve migrar de uma visão baseada apenas na carga de estimulação para uma estratégia de medicina de precisão, com vigilância ecocardiográfica direcionada à detecção precoce de dilatação atrial, RM e remodelamento estrutural progressivo, também em pacientes com DNS.

O artigo contribui de forma significativa para a Literatura ao demonstrar que DNS e BAV, embora compartilhem benefícios sintomáticos semelhantes com o uso do MP, apresentam trajetórias distintas de remodelamento cardíaco e fatores de risco para mortalidade. Estudos prospectivos e multicêntricos, com avaliação de estratégias de estimulação alternativas, são necessários para validar e expandir essas recomendações.^11,12^
