# Supplementary material for: Além do Pacing Ventricular: A Cardiomiopatia Atrial como Determinante da Evolução Clínica após Implante de Marca-passo?
Source: Arq Bras Cardiol. 2026 Jul 2;123(5):e20260425. [Article in Portuguese] doi: 10.36660/abc.20260425 (PMC13399342; doi:10.36660/abc.20260425)
Supplement: Translation

# Translation: Beyond Ventricular Pacing: Is Atrial Cardiomyopathy a Determinant of Clinical Outcome after Pacemaker Implantation?

**DOI:** 10.36660/abc.20260425i

**Authors:** Bianca Lopes Cunha, Helena Nogueira Brasil, Patrícia de Araújo Matias, Eduardo Arrais Rocha

**Affiliations:** 1 Fortaleza CE Brazil Universidade Federal do Ceará, Fortaleza, CE – Brazil; 2 Fortaleza CE Brazil Hospital de Messejana, Fortaleza, CE – Brazil; 3 Fortaleza CE Brazil Centro de Arritmia do Ceará (CACE), Fortaleza, CE – Brazil; 4 Fortaleza CE Brazil Universidade Federal do Ceará Hospital Universitário Walter Cantídio, Fortaleza, CE – Brazil; 5 Fortaleza CE Brazil Unichristus, Fortaleza, CE – Brazil

**Keywords:** Artificial Pacemaker, Cardiomyopathies, Ventricular Remodeling

The study "Clinical Outcomes Following Pacemaker Implantation: A 3-Year Comparative Study of Sick Sinus Syndrome and Atrioventricular Block"^1^ presents a relevant comparative analysis between patients with sick sinus syndrome (SSS) and atrioventricular block (AVB) who underwent permanent pacemaker implantation and were followed for three years. The main merit of the work lies in the clinical evaluation and assessment of cardiac remodeling, atrial fibrillation (AF) rates, readmissions, and mortality compared between the two groups.

Among the study's strengths are the relatively long follow-up period, with serial echocardiographic evaluation, showing important differences between the patterns of cardiac remodeling. Patients with SSS showed a progressive increase in left atrial volume (LAV) and left ventricular end-diastolic diameter (LVDD), in addition to a significant worsening of mitral regurgitation (MR). In contrast, patients with left atrial block (LAB), even with a ventricular pacing load above 90%, showed relative stability in atrial and ventricular dimensions. Both groups had slight reductions in ejection fraction (EF), high rates of AF, hospitalizations, and mortality, without significant differences between the groups. These findings partially challenge the classic concept that right ventricular pacing alone is the main cause of cardiac remodeling.

Cardiac remodeling refers to molecular, cellular, and interstitial changes that alter the size, shape, and function of the heart after injury or chronic overload.^2^ Clinically, it manifests as chamber dilation and deterioration of systolic function, constituting an important substrate for heart failure (HF) and arrhythmias.

This work also deserves recognition for discussing potential prognostic differences between SSS and AVB. While MR was associated with mortality in SSS, in AVB, the predictors were coronary artery disease, use of beta-blockers, and increased LAV. These findings suggest distinct pathophysiological mechanisms and reinforce the need for rigorous follow-up of these patients.

The high incidence of AF in both populations, exceeding 35% in three years, reinforces observations previously demonstrated in studies such as ASSERT^3^ and MOST,^4^ in which patients with cardiac devices presented a high incidence of subclinical atrial arrhythmias and a subsequent increase in thromboembolic risk.

However, some important methodological limitations, although cited in the study, need to be considered. The study was retrospective, single-center, and observational, which reduces its ability to establish robust causal relationships. The number of patients was relatively small, especially in the SSS group, limiting more consistent multivariate analyses. Furthermore, there was no detailed assessment of ventricular electrode position or the occurrence of ventricular or atrioventricular dyssynchrony.

The lack of more complete data on HF and pacing-related cardiomyopathy limits the analysis. Classic studies such as DAVID,^5^ MOST,^4^ and BLOCK-HF^6^ have already demonstrated that high right ventricular pacing loads can promote ventricular dysfunction and increased hospitalization for HF. Interestingly, in this study, patients with AVB and pacing loads above 90% showed no differences in EF and paradoxically had smaller changes in cardiac remodeling. This observation may reflect baseline differences in atrial and myocardial substrate, but it may also result from statistical limitations and echocardiographic analyses, considering different analyzers and the absence of revalidation of echocardiographic data, the absence of atrial and ventricular strain analysis, as well as data on the positions of the ventricular and atrial electrodes.

The authors suggest that SSS itself could represent an independent marker of remodeling. This hypothesis is biologically plausible, especially considering the growing recognition of atrial cardiomyopathy as a clinical entity. Recent studies^7,8^ demonstrate that atrial fibrosis, inflammation, degeneration of the conduction system, and structural changes in the atrium precede both AF and SSS manifestations. In this context, atrial cardiomyopathy may have been one of the main pathophysiological determinants of the differences observed between the groups in this study. These findings directly challenge the notion that ventricular stimulation alone is the main determinant of adverse remodeling.

These findings directly relate to more modern concepts of atrial cardiomyopathy, in which the left atrium is no longer seen merely as an indirect marker of cardiovascular disease but is understood as an active component of the cardiovascular pathophysiological process. This interpretation further strengthens the rationale for early echocardiographic monitoring strategies, surveillance of subclinical AF, and the eventual adoption of physiological atrial pacing modalities in patients with subclinical AF.

On the other hand, programming excessively long AV intervals in SSS, to avoid ventricular stimulation in patients with already compromised atrioventricular conduction, can lead to hemodynamic impairments, such as diastolic MR, resulting in the appearance of symptoms and/or AF.^9^

From a clinical standpoint, the study reinforces the need for periodic echocardiographic follow-up of pacemaker (PM) patients, particularly those with SSS. Thus, the percentage of pacing should be interpreted not only as a marker of device dependence but also as an expression of the underlying pathophysiology. While in the AVB, the traditional concern remains focused on pacing-induced ventricular dyssynchrony, in SSS, the greatest challenge appears to be the silent progression of structural atrial and ventricular disease.

Thus, the study findings and contemporary guidelines^9,10^ suggest that post-implant follow-up should shift from a view based solely on pacing load to a precision medicine strategy, with echocardiographic surveillance aimed at the early detection of atrial dilation, MR, and progressive structural remodeling, also in patients with SSS.

This article makes a significant contribution to the literature by demonstrating that SSS and AVB, while sharing similar symptomatic benefits with the use of PM, present distinct trajectories of cardiac remodeling and risk factors for mortality. Prospective and multicenter studies, evaluating alternative pacing strategies, are needed to validate and expand these recommendations.^11,12^
